# Uniformity of cardiac ^123^I-MIBG uptake on SPECT images in older adults with normal cognition and patients with dementia

**DOI:** 10.1007/s12350-019-01977-5

**Published:** 2019-12-09

**Authors:** Gemma Roberts, Jim J. Lloyd, Elizabeth Jefferson, Joseph P. M. Kane, Rory Durcan, Sarah Lawley, George S. Petrides, Kim Howe, Iftikhar Haq, John T. O’Brien, Alan J. Thomas

**Affiliations:** 1 Institute of Neuroscience, Newcastle University, Biomedical Research Building, Campus for Ageing and Vitality, Westgate Road, Newcastle upon Tyne, NE4 6BE UK; 2 Nuclear Medicine Department, Leazes Wing, Royal Victoria Infirmary, Richardson Road, Newcastle upon Tyne, NE1 4LP UK; 3 Centre for Public Health, Queen’s University Belfast, Royal Victoria Hospital, Belfast, BT12 6BA UK; 4 Cardiology Department, Royal Victoria Infirmary, Richardson Road, Newcastle upon Tyne, NE1 4LP UK; 5 Department of Psychiatry, University of Cambridge, Box 189, Level E4 Cambridge Biomedical Campus, Cambridge, CB2 0SP UK

**Keywords:** Cardiac MIBG, SPECT, sympathetic innervation, cardiac MIBG uptake homogeneity, SPECT artifacts

## Abstract

**Introduction:**

Some studies report that assessing regional ^123^I-cardiac MIBG uptake can aid in the diagnosis of Lewy body disease, but others report heterogeneity in healthy controls. We aimed to evaluate regional cardiac MIBG uptake patterns in healthy older adults and patients with dementia.

**Methods:**

31 older adults with normal cognition, 15 Alzheimer’s disease (AD), and 17 Dementia with Lewy bodies (DLB) patients were recruited. 5 individuals had previous myocardial infarction. Participants with sufficient cardiac uptake for regional SPECT analysis (29/31 controls, 15/15 AD, 5/17 DLB) had relative uptake pattern recorded. Controls were assessed for risk of future cardiovascular events using QRISK2, a validated online tool.

**Results:**

In controls uptake was reduced in the inferior wall (85%), apex (23%), septum (15%), and lateral wall (8%). AD and DLB showed similar patterns to controls. Lung or liver interference was present in 61% of cases. Myocardial infarction cases showed regional reductions in uptake, but normal/borderline planar uptake. In controls, there was no relationship between cardiovascular risk score and uptake pattern.

**Conclusions:**

Significant variability of regional cardiac ^123^I-MIBG uptake is common in cases with normal planar cardiac uptake. Heterogeneity of regional uptake appears non-specific and unlikely to aid in the diagnosis of Lewy body disease.

**Electronic supplementary material:**

The online version of this article (10.1007/s12350-019-01977-5) contains supplementary material, which is available to authorized users.

## Background

Dementia with Lewy bodies (DLB) is the second most common form of neurodegenerative dementia after Alzheimer’s disease, accounting for 5-10% of cases.^1^,^2^ Accurate diagnosis is important for clinical management, prognosis, and carer wellbeing,^3^–^5^ but initial misdiagnosis outside the specialist setting is common.^2^,^6^,^7^ Cardiac ^123^I-metaiodobenzylguanidine sympathetic innervation imaging (“cardiac MIBG”) is an established technique for differentiating DLB from other dementias. Sympathetic denervation occurs in DLB, resulting in low cardiac MIBG uptake on the scan, whereas healthy controls and patients with other dementias such as Alzheimer’s disease have normal uptake.^8^–^14^ The technique has been used in Japan in Lewy body disease for over a decade and was recently included as an indicative biomarker in the fourth DLB consensus criteria, alongside ^123^I-FP-CIT SPECT.^6^

Cardiac MIBG studies are typically quantified on planar images using the heart-to-mediastinum ratio (HMR) as a diagnostic indicator, where HMR is the ratio between the count density in a cardiac left ventricular region (mean pixel value) and the count density in a mediastinal region of non-specific uptake. Abnormal scans have reduced cardiac uptake as indicated by a low HMR. The HMR is an assessment of the overall cardiac uptake and does not take regional differences in uptake throughout the left ventricle into account. Regional variation of uptake can be assessed using SPECT imaging.

Published studies exploring the use of SPECT cardiac MIBG imaging for diagnosing Lewy body disease have suggested that regional assessment of cardiac uptake could improve sensitivity as regional defects may occur before global loss is apparent.^13^,^15^–^19^ However, we have noted that regional count reductions are sometimes seen in probable AD subjects who have normal planar scan appearances, suggesting that discrete areas of reduced regional counts may be non-specific, e.g., due to unreported silent myocardial infarction, or artifactual, e.g., due to attenuation or the influence of extra-cardiac activity. It has been reported in the literature that heterogeneous cardiac MIBG count distribution can occur in healthy controls, with relative reduction most commonly seen in the inferior wall.^19^–^26^ This may be due to true physiological variation in sympathetic nerve distribution or artifacts caused by the imaging process—either from the signal being attenuated by the body or by interference from non-cardiac activity, predominantly the liver.^27^–^29^ Although several groups investigating the diagnostic accuracy of cardiac ^123^I-MIBG for the diagnosis of Lewy body disease have acquired SPECT data alongside planar, often it is only the planar scans that have been used for analysis with the SPECT results not reported,^12^,^30^–^33^ suggesting that the added value of SPECT is unclear. In their 2015 multicentre study, Yoshita et al used SPECT for visual analysis but it was not clear on what basis the SPECT images were deemed normal or abnormal.^10^

We recruited volunteers over 60 years of age, healthy controls, DLB patients, and Alzheimer’s disease patients, and performed cardiac MIBG SPECT imaging to gain a better understanding of cardiac MIBG distribution in these groups and therefore how to interpret patient scans. Our objective was to compare the distribution pattern of MIBG in the heart seen in these clinical groups. The underlying hope was that we may be able to distinguish confounding regional reductions in cardiac uptake due to cardiac disease from pathological ones due to Lewy body disease by looking at heterogeneity, thereby improving specificity. Sensitivity may also be improved if we were to find any characteristic patterns of reduction associated with Lewy body disease.

## Methods

### Participants

#### Older people without cognitive impairment (controls)

Thirty-one healthy adults aged over 60 years were recruited as part of an ongoing study into cardiac MIBG in a representative UK population of older adults. They underwent a detailed neurological and cognitive examination by a research physician. All had had normal cognition, no parkinsonism, and had a normal MRI brain scans. No subjects had Class II or worse heart failure according to the New York Heart Association classification,^34^ or had had a myocardial infarction in the previous year or were taking tramadol, labetalol or tricyclic antidepressants. Previous cardiac history, such as myocardial infarction (MI) more than 12 months prior to recruitment or atrial fibrillation was recorded as part of medical history taking. Subjects were not excluded if they had risk factors for cardiac disease, such as a history of smoking, raised blood pressure, or Type 2 diabetes because we aimed to recruit typical older adults with normal cognition. In our clinical studies in DLB we aim to assess cardiac MIBG in a “real-world” setting, where risk factors for cardiac disease are extremely common. However, a detailed medical history was taken, which later enabled an estimate of cardiac risk to be made using the QRISK2 tool, recommended by NICE in Clinical Guideline 181 Cardiovascular disease: risk assessment and reduction.^35^ QRISK2 uses the ratio of total cholesterol to high density lipoproteins as a risk factor, with a ratio of 4 used as default. These parameters were not part of our original study design so were not available for most of the volunteers. As the true ratios were not known, a ratio of 6, which is regarded as the upper limit by Heart UK, was input for those with high cholesterol disclosed in their medical history and the default of 4 used for everyone else. Two subjects had documented heart disease, making the QRISK2 score inappropriate—their risk scores were still calculated with this recorded as a limitation. QRISK2 scores in controls were assessed for any correlation with cardiac uptake using linear regression.

#### Older people with dementia

Seventeen patients meeting the clinical criteria for probable dementia with Lewy bodies and 15 meeting criteria for dementia due to Alzheimer’s disease were recruited from memory clinics, as described in our previous publications.^36^,^37^ Briefly, patients had their dementia diagnoses confirmed by an expert panel of old-age psychiatrists. They were categorized as having probable DLB if two or more of the core consensus criteria for DLB 6 (fluctuations, visual hallucinations, REM sleep behavior disorder and parkinsonism) were present, and AD if none of these were present. The same broad general inclusion criteria as discussed above for the controls were applied to ensure the patients were typical of the local population. Cardiac risk was not calculated for the dementia patients since their cardiac uptake was expected to be affected by Lewy body disease in many cases. However cardiac history was recorded to enable comparison with SPECT imaging results. We have previously showed no convincing evidence of lower cardiac MIBG uptake in patients with a previous MI, although this was a small subset.^36^

### Image Acquisition

All participants were administered 111 ± 10% MBq I-123-MIBG via slow intravenous injection. Potassium iodate tablets (170 mg) were given before and after injection to minimize uptake of free iodine by the thyroid. Images were acquired on a dual headed Siemens Symbia Intevo or Siemens Symbia T series gamma camera (Siemens Healthcare, Munich, Germany) with medium energy low penetration (MELP) collimators. Ten minute anterior planar images were acquired at 4 h (± 30 min) after injection. SPECT imaging was carried out immediately after the planar image with the subject in the supine position with arms raised if possible; however the majority of controls (19/31) and patients (7/15 AD; 13/17 DLB) were scanned with their arms by their sides. Due to patient comfort it was not possible to complete SPECT imaging, even with arms down, for two of the 17 DLB patients, both of whom had low uptake on planar imaging. For planar imaging the energy window was 159 keV ± 10%, matrix size was 128 × 128 and no zoom was applied. SPECT images were acquired in H mode over 360 degrees with 120 projections of 20 s using a non-circular autocontoured orbit. The energy window was 159 keV ± 7.5%, matrix size 64 × 64, and zoom 1.64.

### Image Processing

All images were processed on a Hermes workstation (Hermes Medical Ltd, Stockholm). Delayed anterior planar images were analyzed to obtain the heart-to-mediastinum ratio (HMR) using a 6 cm circular ROI placed over the left ventricle and 4 × 3 cm rectangular ROI between the lungs in the mediastinum as described in our previous publication.^37^ SPECT images were processed in Hermes Hybrid recon using filtered back projection with a Butterworth filter of 0.38 cycles per cm cut-off and order 8 applied. The images were reoriented during reconstruction to align with the horizontal and vertical long axes of the myocardium, as is standard for myocardial perfusion SPECT, with as much extra-cardiac activity (e.g., liver and lung) masked out as possible.

### Image Assessment

Quantitative perfusion SPECT (QPS) software (Hermes Medical Ltd, Stockholm) was used to display the short axis, vertical long axis, and horizontal long axis slice images in rows for visual assessment of relative uptake. Polar plots of relative uptake pattern were also reviewed. These plots display the average relative uptake in the apex, anterior, lateral, inferior, and septal walls as a percentage of the maximum pixel value. The images and polar plots were reviewed blinded to the clinical groupings by a Consultant Clinical Scientist experienced in using the software for myocardial perfusion imaging (EJ). The overall degree of uniformity and the relative uptake for each of the left ventricle walls (anterior, inferior, septal, lateral, and the apex) was recorded using the categories given in Table 1. The ratings categories were developed by GR, EJ and JJL by reviewing cardiac MIBG images not included in the current study.

**Table 1:** Rating categories for overall assessment of uptake pattern in the left ventricle and of uptake in each of the ventricular wall regions

Overall rating categories	Uptake categories for each wall	Interference categories
Not possible to score due to interference Definitely uniform Very slight heterogeneity Patchy Clear defect Multiple clear defects Other - see comment	Normal Mildly reduced/patchy Moderately reduced Severely reduced No uptake	No significant interference Liver interference Lung interference Both liver and lung interference

## Results

### Controls with Normal Cognition

Of the 31 controls recruited, 2 had to be excluded due to their image appearances (see below). The remaining 29 people included were aged between 62 and 94 years (mean 75.2). Most were male (22/29) and white (28/29). Body mass indices (BMI) ranged from 22 to 38 kg/m^2^, with the mean value of 27.2 in the overweight category. These demographics are summarized in the controls and dementia patients are taken from concurrent clinical studies, so not all the same information was collected for dementia patients.

Table 2 alongside incidence of cardiovascular risk factors such as smoking. The controls and dementia patients are taken from concurrent clinical studies, so not all the same information was collected for dementia patients.

**Table 2:** Demographics summary for the controls and patients with DLB and AD included in the study

	Controls	DLB	AD
Age			
Mean ± SD	75.2 ± 8.3	77.5 ± 8.0	76.2 ± 6.8
Range	62 to 94 years	60 to 89 years	62 to 85 years
Sex	7 female (24%)	2 female (12%)	4 female (27%)
	22 male (76%)	15 male (88%)	11 male (73%)
BMI (kg/m^2^)			
Mean ± SD	27.8 ± 4.2	Not measured	Not measured
Range	21.6 to 37.8		
Ethnicity	28 white (97%)	Not recorded	Not recorded
	1 Asian (3%)		
Smoking	13 never smoked (45%)	Not measured	Not measured
	13 ex-smokers (45%)		
	2 current smokers (7%)		
	1 not recorded (3%)		
Diabetes	0 Type I (0%)	Not recorded	Not recorded
	3 Type II (10%)		
Systolic blood pressure			
Mean ± SD	140.5 ± 17.8	Not recorded	Not recorded
Range	112 to 173		
On blood pressure medication	8 (28%)	4 (24%)	5 (33%)
Hypercholesterinaemia documented	11 (34%)	Not recorded	Not recorded
Documented cardiac history	Previous MI and AF: 1 (3%)	Previous MI: 2 (12%)	Previous MI: 3 (20%)
	Angina: 1 (3%)		
	ECG changes: 1 (3%)		

**Figure 1:**
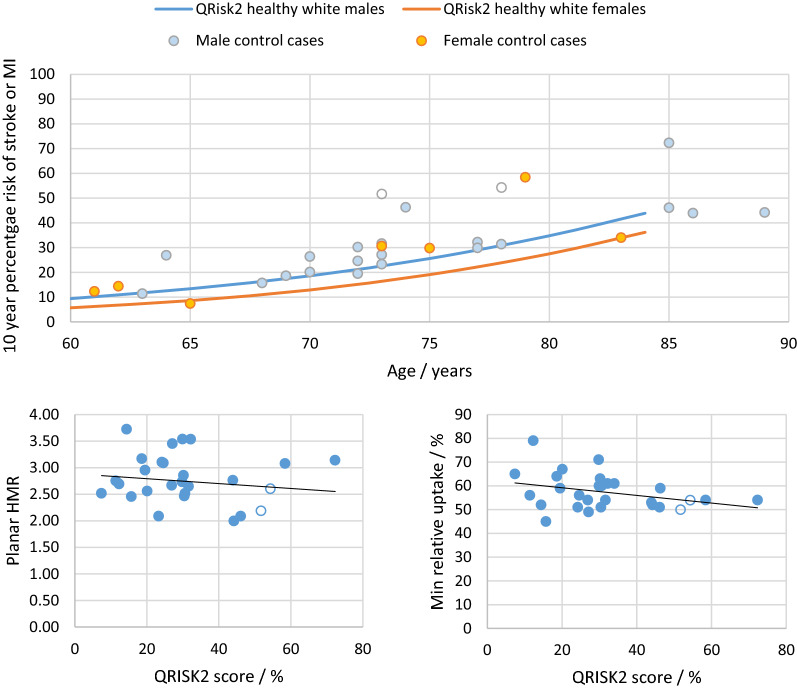
Top: Plot of cardiac risk for our control subjects against subject age for males (blue data points) and females (orange data points. The two males with previous cardiac history are shown in open circles. The solid lines correspond to the mean QRISK2 database scores for white males and females with no cardiac risk factors. The database is only available up to 84 years. Bottom: Plots of HMR and minimum relative uptake against cardiac risk. Controls with previous cardiac history are shown in open circles

Ten-year cardiac risk scores assessed with QRISK2 showed the group to be at high risk, as expected for older adults (Figure 1). Most subjects were at higher risk than expected for age due to the inclusion of one or more cardiac risk factors in the calculation. However, despite slight downward trends, there was no significant correlation between cardiac risk and either overall cardiac MIBG uptake as given by planar HMR (*P* = .64) or minimum percentage relative uptake on SPECT (*P* = .08) (Figure 1).

Two controls had abnormally low uptake on planar imaging (below the normal threshold of 2.2 suggested for medium energy collimators by the Japanese normal database of controls^20^) and no discernible uptake on SPECT imaging. These individuals had no significant medical history and were not taking any medications suspected of interfering with cardiac MIBG uptake, including those without strong clinical evidence of an effect.^38^ It is well recognized that occult Lewy body disease (usually termed incidental Lewy body disease) is present in many older people,^39^ which may explain the findings in controls.

**Figure 2:**
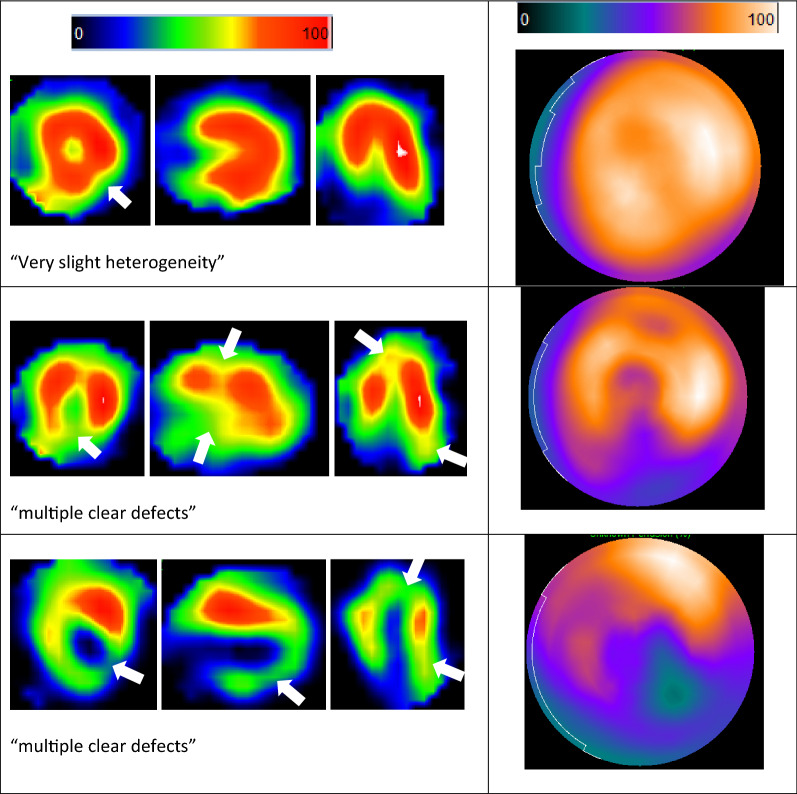
Top: QPS slice data and polar plot for the single control rated as within normal limits, showing almost perfectly uniform MIBG distribution, similar to that seen in normal Tc-99 m myocardial perfusion images. Middle: Slice data and polar plot for a more typical control, with reduced relative MIBG uptake inferiorly and at the apex. Bottom: A very abnormal control but without prior known cardiac events. The sites of reduced relative uptake are shown with white arrows

Of the remaining 29 controls, the mean planar HMR was 2.78 and standard deviation 0.46. The SPECT images for three of the controls were not possible to assess due to a high degree of lung interference. A further two with significant lung interference and two with liver interference were assessed but may be unreliable. Overall, 62% of the controls had some degree of interference; lung interference in 11/29 cases (38%) and liver interference in 7/29 (24%). Almost all controls assessed (25/26) displayed at least one area of clearly reduced relative uptake, most often in the inferior wall, which was rated as normal or mildly reduced in only 4/26 cases (15%). Some example images and ratings categories are shown in Figure 2. The results are summarized in Table 3 alongside those of the dementia patients.

**Table 3:** Frequency of regions showing reduced relative uptake for control, AD, and DLB groups

	Apex	Anterior wall	Lateral wall	Inferior wall	Septal wall
Controls	6/26 (23%)	0	2/26 (8%)	22/26 (85%)	4/26 (15%)
AD	5/15 (33%)	0	2/15 (13%)	13/14 (93%)	4/15 (27%)
DLB	1/5 (20%)	0	0	4/5 (80%)	1/5 (20%)

### People with Dementia

**Figure 3:**
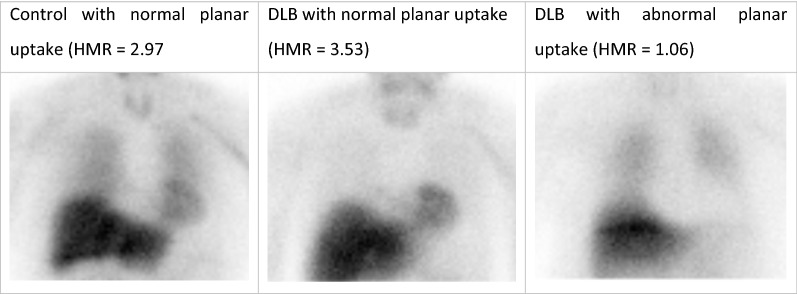
Example planar images of a control, a DLB patient with normal uptake on planar imaging, and a DLB patient with typical lack of cardiac uptake on planar imaging

Of the 15 people with DLB who completed both planar and SPECT imaging, the mean planar HMR was 1.74 ± 0.83, with 10 scans resulting in HMR values below 2.2. One DLB participant with low planar HMR had visible cardiac uptake on SPECT images, but it was too unclear for a meaningful SPECT reconstruction to be obtained. Figure 3 shows example planar images for DLB patients with normal and abnormal cardiac uptake on planar imaging, alongside an example control image. All five of the DLB subjects with normal planar cardiac uptake had uptake visible on SPECT, i.e., the normal HMRs were not caused by overlapping liver or lung. There were therefore only five DLB SPECT scans included for SPECT regional analysis, those with normal planar imaging. Of the 15 people with AD, three had HMR values below 2.2 (mean HMR 2.51 ± 0.36); however, all 15 AD patients had sufficient cardiac uptake to reconstruct clear SPECT images.

The pattern of SPECT uptake was variable but overall similar to that seen in the controls for both AD and DLB (Tables 3, 4).

**Table 4:** Frequency of each overall rating category for control, AD, and probable DLB groups

	Control (n = 29)	AD (n = 15)	DLB (n = 5)
Interference present?	18 (62%)	10 (67%)	2 (40%)
Not possible to score due to interference	7 (24%)	3 (20%)	1 (20%)
Definitely uniform	0	0	0
Very mild heterogeneity	1 (3%)	0	0
Patchy	0	1 (7%)	0
Clear defect	8 (28%)	3 (20%)	2 (40%)
Multiple clear defects	13 (45%)	8 (53%)	2 (40%)

### Effect of Myocardial Infarction on Planar and SPECT Images

**Figure 4:**
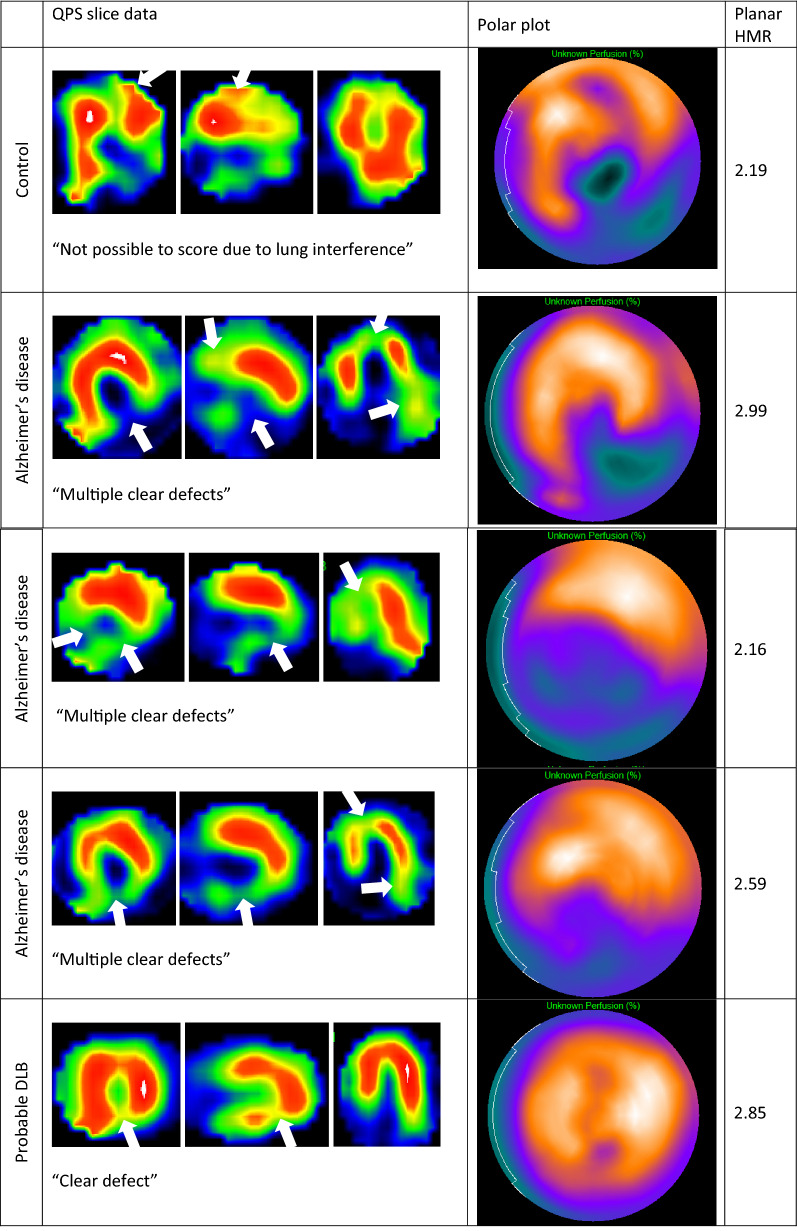
Slice data and polar plots for cases with previous MI, with corresponding planar HMRs. The sites of interference (top image) and relative reduced uptake are shown with arrows

One control, three of the AD patients and two of the DLB patients had had a previous MI. Their images are displayed in Figure 4. The control had a borderline HMR of 2.19 (assuming a normal range > 2.2) despite a large area of apparently reduced uptake at the apex and inferiorly (lung interference anteriorly means this should be interpreted with caution). Of the AD MI cases, one had a borderline HMR of 2.16 and two had normal uptake. Of the DLB MI cases, one had normal HMR (2.85) and one had no visible cardiac uptake and so was one of the cases excluded from SPECT regional analysis.

The cases with previous MI showed defects of varying magnitude and extent on SPECT and variable planar HMRs, none of which were abnormally low. The number of cases is too low to establish a correlation between the extent of regional denervation and effect on global HMR but it seems likely that regional denervation would have to be widespread for planar HMR to be abnormally low.

## Discussion

The majority of the controls (25/26) and all the AD patients showed at least one region of reduced relative cardiac MIBG uptake, with 45% of controls and 53% of AD patients showing relative reduction in multiple regions. This suggests that the presence of areas of reduced uptake on SPECT should not be used to diagnose Lewy body disease, as some authors have suggested, as this is likely to be highly non-specific. Our controls were almost all at high risk of coronary events over the next 10 years, so cardiac disease as a cause for the relative reduction cannot be entirely excluded. However, we note the lack of correlation between QRISK2 score and non-uniformity index suggesting the most at risk individuals are not at significantly greater risk of cardiac sympathetic denervation.

None of the control or dementia cases demonstrated reduced relative uptake in the anterior wall, which at first glance suggests that this region could be a useful target area in which to assess uptake in cases where the planar HMR is suspected of being affected by regional defects. However, 11 of the 29 control SPECT images were difficult to assess due to lung interference, three of whom were impossible to rate, making the anterior wall uptake unreliable.

The strengths of this study include the inclusion of older people thoroughly assessed to have normal cognition but who also had other morbidities associated with aging, including cardiac risk factors, making them a representative sample of older adults. The patients with dementia had an expert panel diagnostic review by three old-age psychiatrists, a method which has been validated against autopsy diagnosis and supported by regulatory authorities as a ‘gold standard’.^40^,^41^ However, a limitation is the use of only one rater for the SPECT uniformity assessments. This does however reflect clinical practice in radionuclide image reporting and the results are convincing even with a single rater. A further limitation is the lack of detailed cardiac assessment and no myocardial perfusion imaging for correlation with cardiac MIBG. However, Simula et al have shown that myocardial perfusion imaging does not correlate with cardiac MIBG SPECT in asymptomatic high coronary risk cases with mild coronary artery stenosis.^42^ They found a link between reduced uptake on cardiac MIBG SPECT and degree of stenosis for the left anterior descending artery (but not the left circumflex or right coronary artery) and suggest that CAD could be the cause of regional inhomogeneities on cardiac MIBG SPECT previously reported as normal variants.^42^ However, on our SPECT scans the areas showing reduced uptake are the inferior wall and/or apex for the majority of controls (21/26), which is against cardiac disease being the main cause as this would be expected to affect any of the cardiac territories. The pattern that we see suggests that either attenuation and scatter are causing this artifact, artifacts have been introduced in acquisition or reconstruction (e.g., due to high liver uptake), or there is a physiological reason for the apex and inferior wall to have reduced uptake.

The literature on regional sympathetic innervation in healthy controls is quite mixed. Most studies report a heterogeneous distribution within the left ventricle, although they do not agree on the sites of reduced uptake, perhaps related to small sample sizes and different age groups. Early work by Sisson and colleagues in the 1980s on 20 men aged 20-62 years showed a relative reduction at the apex only.^43^ The European guidelines on ^123^I-MIBG cardiac sympathetic imaging note that “normal cardiac MIBG distribution includes a relatively low uptake in the inferior wall, which is more pronounced in the elderly” referencing the 1993 study of Gill et al^27^ in seven young adults (29.4 ± 7.5 years) and eight middle aged, not elderly, adults (53 ± 5.1 years). As well as the inferior reduction Gill et al also reported septal reduction, with no difference between apex and base.^27^ Tsuchimochi et al studied 18 men and 11 women without cardiac diseases aged between 21 and 79 years (mean 42 ± 17 years) and reported that the inferior uptake of MIBG decreased with age in healthy controls, especially in men.^19^ This is important in the context of our study, as all participants were over 60 years of age and most of our controls were men. Image quality was however quite limited compared to modern gamma camera images for both this study published in 1995 and those of Sisson^43^ and Gill.^27^

Our results are similar to those of a more recent study published by Asghar et al in 2017^25^ who reported reduced inferior wall uptake in all 14 healthy controls studied, and suggested that these effects are not predominantly caused by imaging artifacts. However, all our control cases had normal anterior wall uptake, which was not the case in the Asghar study. Interestingly, in their 2012 ^11^C Hydroxyephedrine PET study, Wong et al showed that the lateral wall not the inferior wall shows reduced regional innervation in healthy controls, with relative sparing of anterior and septal walls.^44^

Post-mortem tissue studies e.g.,^45^,^46^ report non-uniform distribution of cardiac innervation, for example in the sinus node; but do not describe the relative distribution in the left ventricle so cannot be correlated with MIBG tracer uptake. However, in their post-mortem study on the distribution of autonomic nerves in the human heart, Kawano et al showed a small but statistically significant reduction in the sympathetic innervation of the inferior wall of the left ventricle compared to the anterior wall.^47^ The reduction is only around 20%, making this unlikely to explain the substantial differences seen in ^123^I-MIBG uptake between the anterior and inferior wall in many controls.

It has been hypothesized that due to its tracer kinetics, ^123^I-MIBG imaging is insensitive to substantial nerve losses in cardiac regions with mild to moderate levels of cardiac denervation and that regional nerve losses need to be relatively severe before a reduction in uptake becomes apparent.^48^ If this is the case it makes the modest physiological differences seen by Kawano et al.^47^ even less likely to explain the cardiac MIBG findings. We note that ^11^C Hydroxyephedrine (HED) has similar kinetics to ^123^I-MIBG, so this doesn’t explain the difference in relative uptake patterns.

This leaves artifacts as a possible contributor to the apparent regional reductions in cardiac MIBG uptake. In a study using an anthropomorphic chest phantom with uniform myocardium activity, the count density within the myocardium was inhomogeneous with both medium and low energy collimators.^28^ There was an overestimation of the measured count density in both the anterior and the septal wall and an underestimation of count density in the inferior wall,^28^ which is in line with typical control studies and which the authors assumed was due to attenuation. However, Gill considered attenuation artifacts an unlikely cause of the reduced inferior uptake in their control series, as myocardial perfusion imaging with thallium-201 did not show corresponding reductions.^27^ We note that although we commonly see mildly reduced relative uptake in the inferior wall on clinical myocardial perfusion images obtained with ^99m^Tc-tetrafosmin, these reductions are typically less obvious than those seen on the majority of our control ^123^I-MIBG images. However, both ^99m^Tc and ^201^Tl have different physical properties ^123^I, particularly in terms of high energy emissions and may not give the same apparent cardiac uptake patterns. This discrepancy could be potentially be resolved with the use of SPECT-CT imaging to correct for attenuation and scatter of the gamma rays from the MIBG tracer within the body. A recent phantom study by Shibutani et al demonstrated an inferior artifact in a myocardium insert (scanned with a low-medium energy general purpose collimator), which resolved with Siemens Flash3D SPECT-CT reconstruction with ACSC.^49^

**Figure 5:**
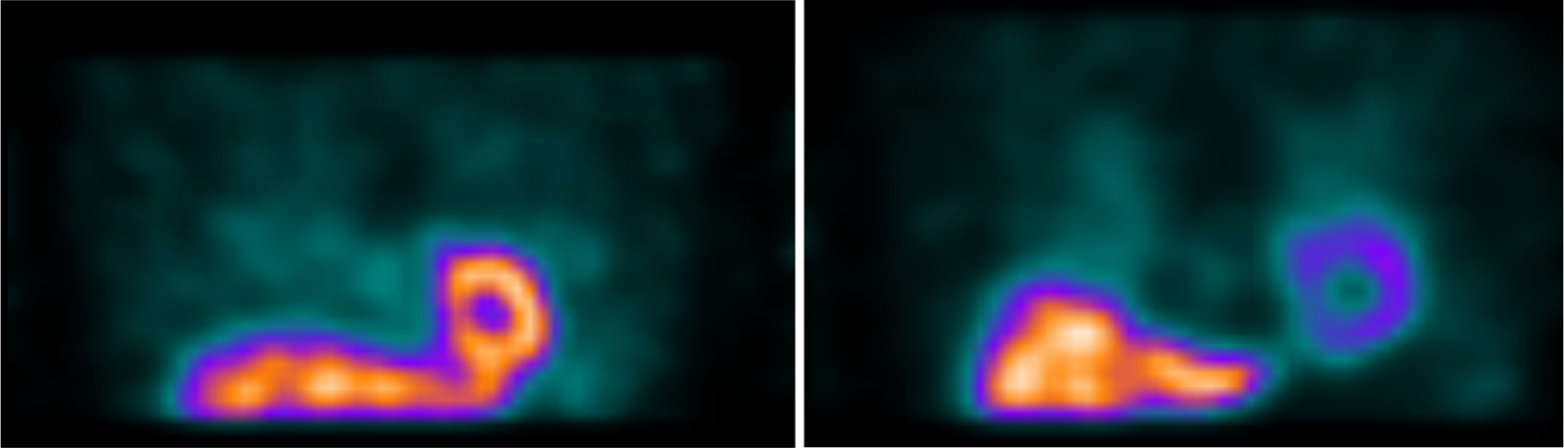
Left: coronal MIBG slice for the control subject with uniform relative cardiac uptake. Right: a more typical control with high liver uptake relative to the heart. Images are displayed scaled to the maximum voxel

Regarding our single control regarded as having uniform relative uptake, the following observations are of note. The planar HMR was 2.69, which is a typical value in line with the other controls (mean HMR 2.78 ± 0.46), rather than an outlier. Similarly, the BMI of this individual was unremarkable at 28.6 (mean 27.8 ± 4.2). However, the relative uptake of the heart is similar to that of the liver, whereas in most of the controls the uptake is much higher in the liver than the heart (Figure 5). In addition, the positioning of the dome of the liver is just inferior to the heart, which is not the case in many of the other controls. This suggests that high liver uptake may be contributing to the apparent reduced inferior wall uptake seen in most of the controls. One of the few studies to directly compare ^11^C Hydroxyephedrine PET with ^123^I-MIBG SPECT, in patients with heart failure and coronary heart disease, reported lower liver uptake on ^11^C Hydroxyephedrine PET than ^123^I-MIBG SPECT, with increased uptake in the inferior and septal regions,^50^ so this may explain the difference in relative distribution reported with the two tracers. A study comparing both tracers in ten healthy young men with normal BMI showed lower relative uptake in the inferior wall and septum with ^123^I-MIBG SPECT compared with ^11^C-HED.^51^ This further suggests that liver uptake may be responsible for the inferior reduction, but we note that attenuation and scatter correction was used for the PET imaging but not the SPECT. To our knowledge, no other studies of ^123^I-MIBG and ^11^C-HED have been carried out in the same cohort of healthy controls and no studies at all with CT attenuation and scatter correction for ^123^I-MIBG. Further research in this area would help to clarify the reasons for the different pattern seen between SPECT and PET.

### Limitations

As discussed above, a limitation of this study is the lack of definitive information on cardiac disease status of the participants. The QRISK2 score gives an idea of the likelihood of future events, but it is impossible to exclude undiagnosed asymptomatic CAD. The patients and controls were recruited to a dementia research study for which detailed cardiac assessment was not part of the study protocol; however an ECG and autonomic dysfunction tests were carried out. A further limitation of this study is the lack of data from younger subjects as only those over 60 years of age were recruited. SPECT-CT data for attenuation and scatter correction were not available for the patients so could not be assessed as part of this analysis.

The administered activity of 111 MBq is lower than reported in some cardiac MIBG studies, with 370 MBq typically used in the US. However, 111 MBq is used in Japan, where the majority of cardiac MIBG imaging studies for dementia diagnosis have been performed. Although the lower activity could give rise to slightly noisier images, it would not explain the clear defects in uptake seen in this study.

Due to the majority of DLB patients having severely reduced cardiac MIBG uptake, it was only possible to study relative uptake in a small group of five subjects with normal planar uptake. However, this group showed similar non-uniform SPECT uptake to the larger control and AD groups. Our results in the AD and control groups suggest that the presence of regional defects in uptake cannot be a specific marker of Lewy body disease. This is the case regardless of the number of DLB cases included.

## New Knowledge Gained

Areas of relatively reduced cardiac ^123^I-MIBG uptake on SPECT images are common, but not ubiquitous, in cognitively normal older adults and patients with dementia who have normal planar cardiac uptake. Uptake is often reduced in the inferior wall and sometimes in the septal wall, lateral wall, and apex. Anterior wall uptake is often influenced by lung interference so appears unsuitable as a target region for uptake assessment, at least on images uncorrected for scatter and attenuation.

## Conclusion

Reduced regional cardiac MIBG uptake in the inferior wall of the left ventricle is common in older adults and most likely related to attenuation or liver uptake. Reductions in other areas may also be artifactual or may be caused by mild underlying coronary artery disease. The pattern of reduction is in any case non-specific and therefore unlikely to aid in the diagnosis of Lewy body disease in typical clinical settings.

## Electronic supplementary material

Below is the link to the electronic supplementary material.

Electronic supplementary material 1 (PPTX 735 kb)

## Abbreviations

DLB: Dementia with Lewy bodies
AD: Alzheimer’s disease
FP-CIT: I-123N-ω-fluoropropyl- 2β-carbomethoxy- 3β-(4-iodophenyl) nortropane
MIBG: I-123 metaiodobenzylguanidine
SPECT: Single photon emission computed tomography
HMR: Heart-to-mediastinum ratio
MI: Myocardial infarction
AF: Atrial fibrillation
CAD: Coronary artery disease
ECG: Electrocardiogram
