# Supplementary material for: Uniformity of cardiac 123I-MIBG uptake on SPECT images in older adults with normal cognition and patients with dementia
Source: J Nucl Cardiol. 2019 Dec 9;28(5):2151–63. doi: 10.1007/s12350-019-01977-5 (PMC8648658; doi:10.1007/s12350-019-01977-5)
Supplement: Supplementary file 1 — Electronic supplementary material 1 (PPTX 735 kb) [file 12350_2019_1977_MOESM1_ESM.pptx]

## Slide 1
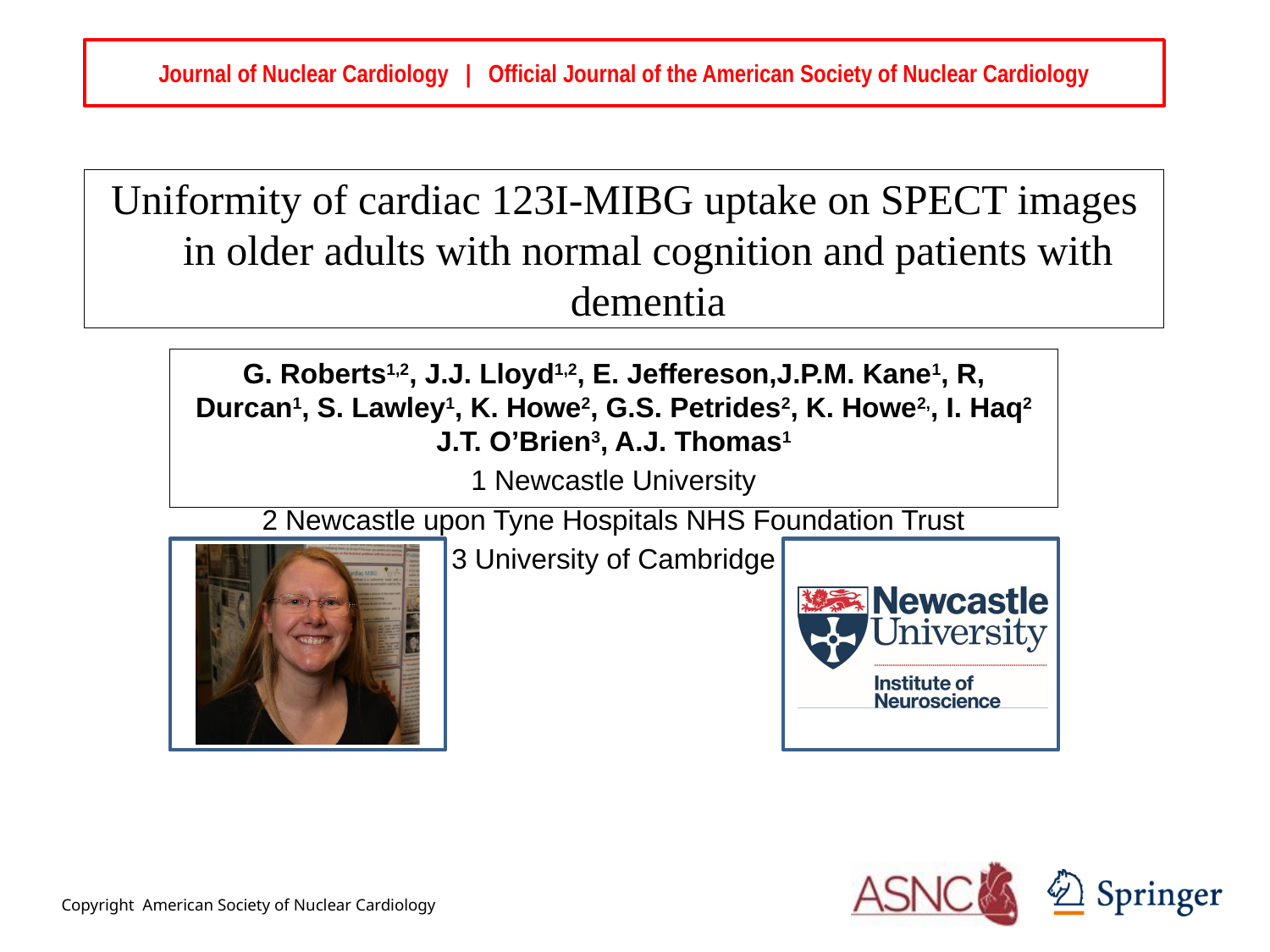

Journal of Nuclear Cardiology | Official Journal of the American Society of Nuclear Cardiology
# Uniformity of cardiac 123I-MIBG uptake on SPECT images in older adults with normal cognition and patients with dementia
G. Roberts1,2, J.J. Lloyd1,2, E. Jeffereson,J.P.M. Kane1, R, Durcan1, S. Lawley1, K. Howe2, G.S. Petrides2, K. Howe2,, I. Haq2 J.T. O’Brien3, A.J. Thomas1
1 Newcastle University
2 Newcastle upon Tyne Hospitals NHS Foundation Trust
3 University of Cambridge
Head shot of author
required
Copyright American Society of Nuclear Cardiology

## Slide 2
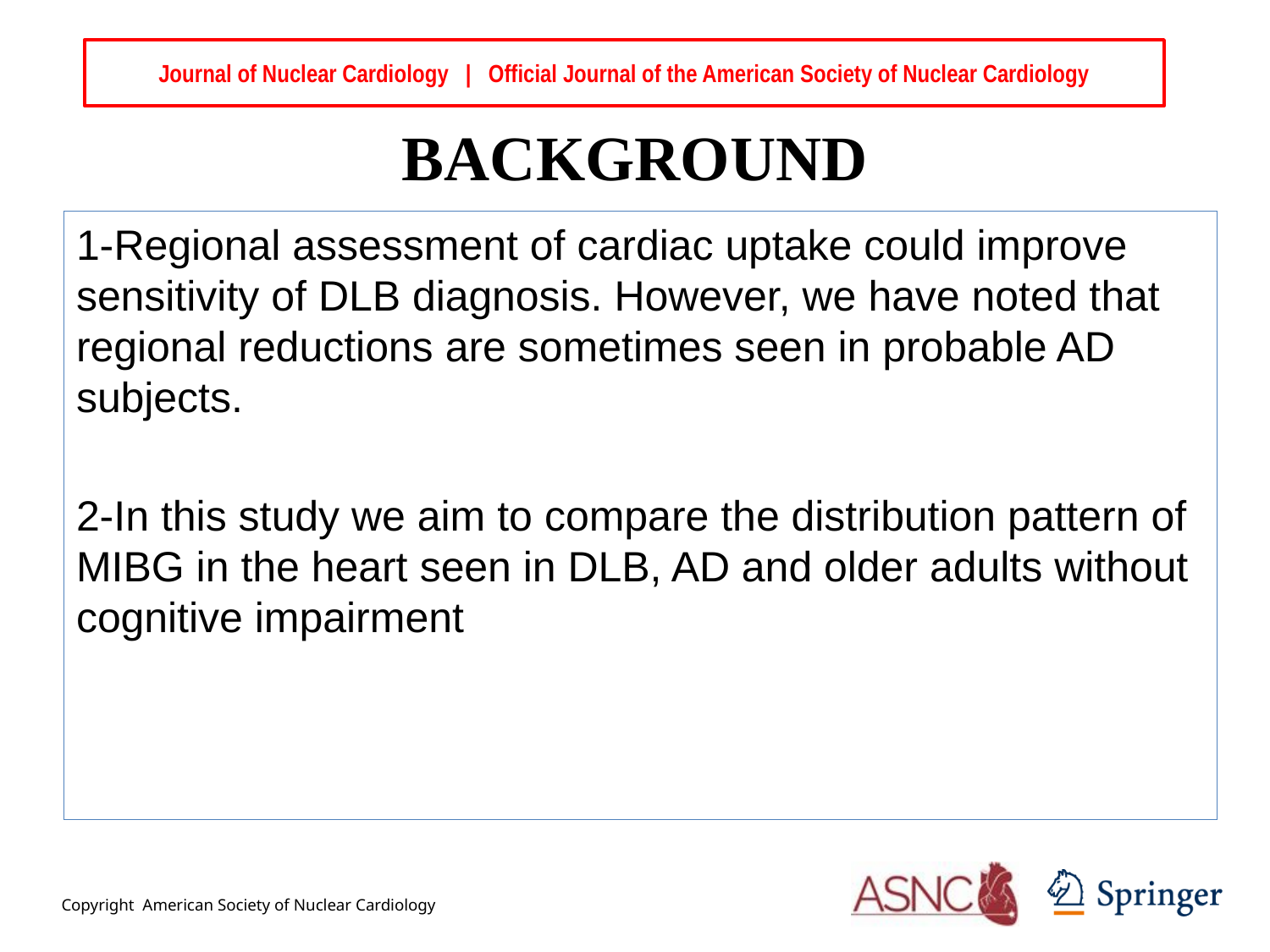

Journal of Nuclear Cardiology | Official Journal of the American Society of Nuclear Cardiology
# BACKGROUND
1-Regional assessment of cardiac uptake could improve sensitivity of DLB diagnosis. However, we have noted that regional reductions are sometimes seen in probable AD subjects.
2-In this study we aim to compare the distribution pattern of MIBG in the heart seen in DLB, AD and older adults without cognitive impairment
Copyright American Society of Nuclear Cardiology

## Slide 3
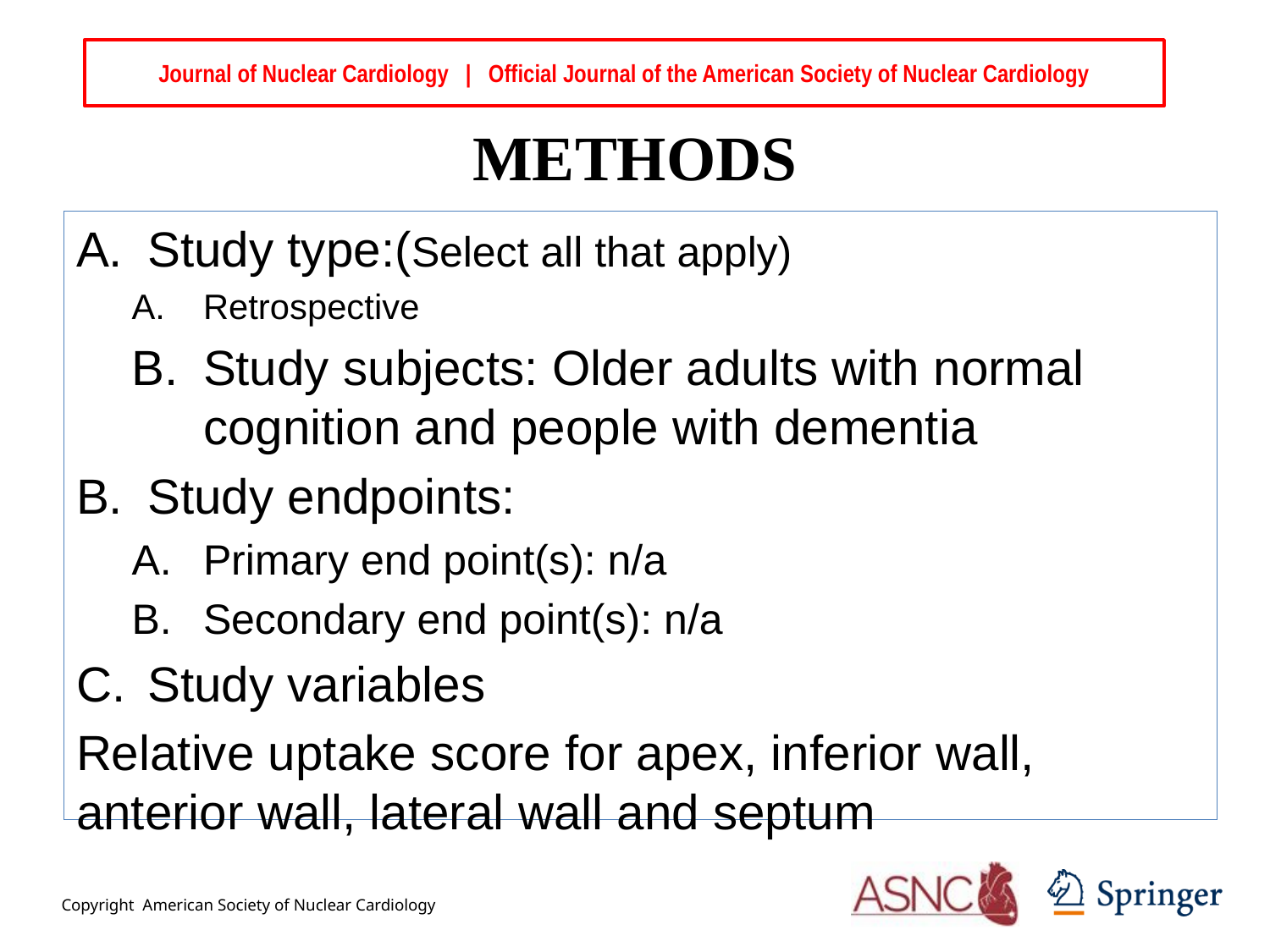

Journal of Nuclear Cardiology | Official Journal of the American Society of Nuclear Cardiology
# METHODS
Study type:(Select all that apply)
Retrospective
Study subjects: Older adults with normal cognition and people with dementia
Study endpoints:
Primary end point(s): n/a
Secondary end point(s): n/a
Study variables
Relative uptake score for apex, inferior wall, anterior wall, lateral wall and septum
Copyright American Society of Nuclear Cardiology

## Slide 4
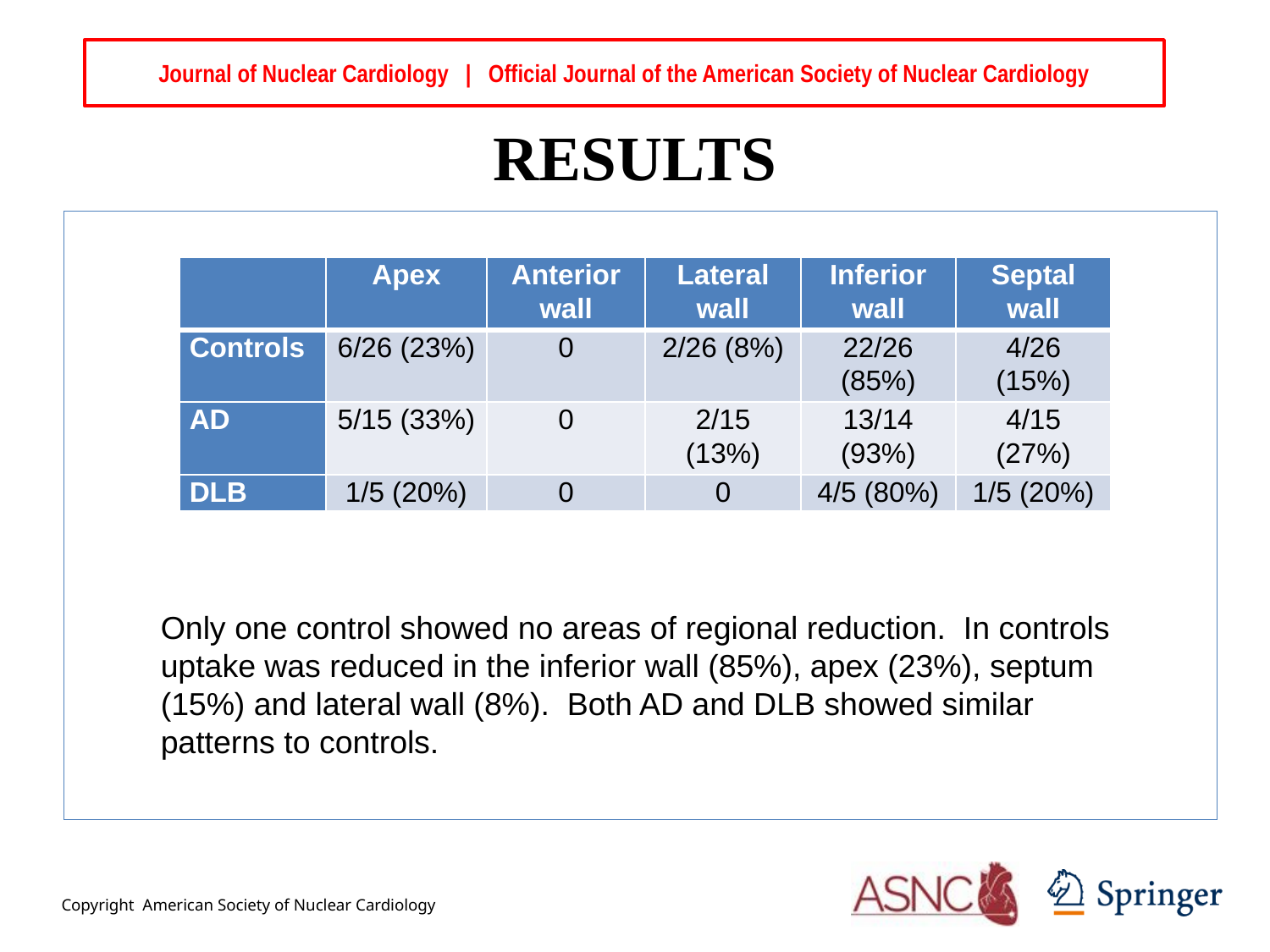

Journal of Nuclear Cardiology | Official Journal of the American Society of Nuclear Cardiology
# RESULTS
| | Apex | Anterior wall | Lateral wall | Inferior wall | Septal wall |
| --- | --- | --- | --- | --- | --- |
| Controls | 6/26 (23%) | 0 | 2/26 (8%) | 22/26 (85%) | 4/26 (15%) |
| AD | 5/15 (33%) | 0 | 2/15 (13%) | 13/14 (93%) | 4/15 (27%) |
| DLB | 1/5 (20%) | 0 | 0 | 4/5 (80%) | 1/5 (20%) |
Only one control showed no areas of regional reduction. In controls uptake was reduced in the inferior wall (85%), apex (23%), septum (15%) and lateral wall (8%). Both AD and DLB showed similar patterns to controls.
Copyright American Society of Nuclear Cardiology

## Slide 5
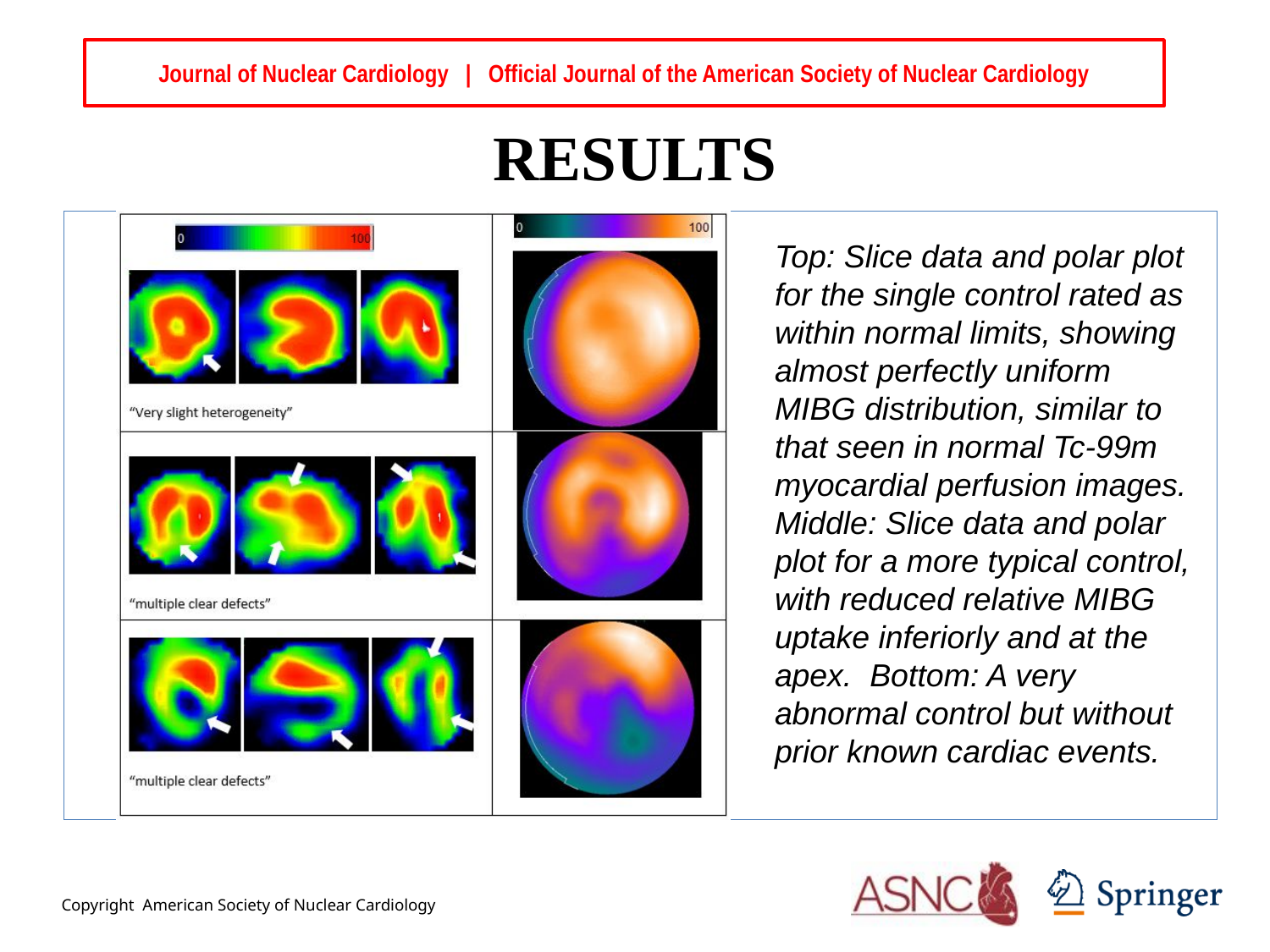

Journal of Nuclear Cardiology | Official Journal of the American Society of Nuclear Cardiology
# RESULTS
Top: Slice data and polar plot for the single control rated as within normal limits, showing almost perfectly uniform MIBG distribution, similar to that seen in normal Tc-99m myocardial perfusion images. Middle: Slice data and polar plot for a more typical control, with reduced relative MIBG uptake inferiorly and at the apex. Bottom: A very abnormal control but without prior known cardiac events.
Copyright American Society of Nuclear Cardiology

## Slide 6
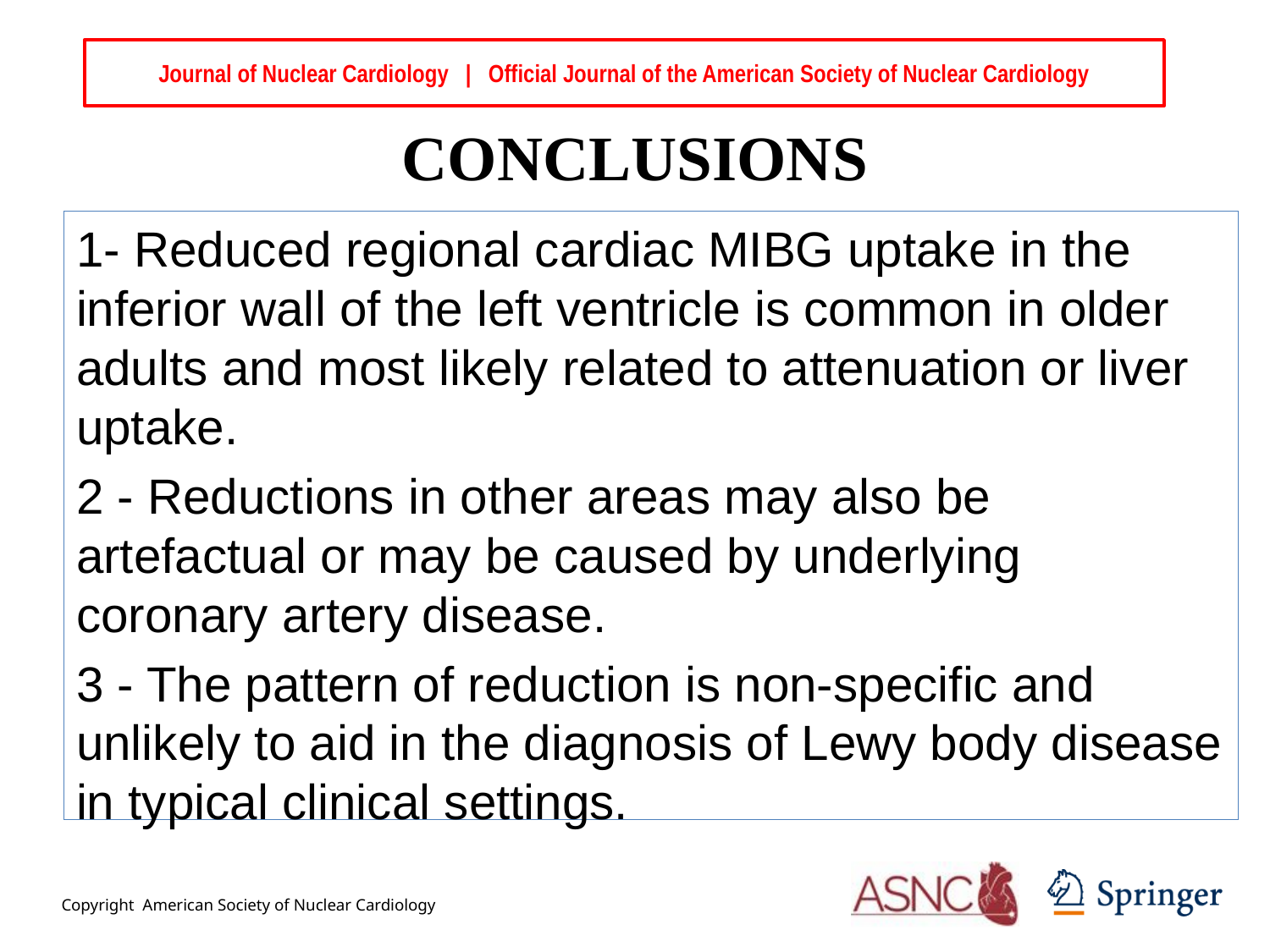

Journal of Nuclear Cardiology | Official Journal of the American Society of Nuclear Cardiology
# CONCLUSIONS
1- Reduced regional cardiac MIBG uptake in the inferior wall of the left ventricle is common in older adults and most likely related to attenuation or liver uptake.
2 - Reductions in other areas may also be artefactual or may be caused by underlying coronary artery disease.
3 - The pattern of reduction is non-specific and unlikely to aid in the diagnosis of Lewy body disease in typical clinical settings.
Copyright American Society of Nuclear Cardiology
